# Dreams

**Published:** 1886-10

**Authors:** 


### HALL'S
### JOURNAL OF HEALTH

VOL. 33. OCTOBER, 1886. No. 10

### *DREAMS.*

"We are such stuff
As dreams are made of, and our little life
Is rounded with a sleep."

We once had a friend whose four score years and ten found him a lonely wanderer upon the verge of the dark river, whose mystic ferryman waits, oar in hand, to conduct to the untried shore beyond, every mortal in his turn. Pausing there and looking back upon his shadowy post, he was wont to exclaim in a tone, saddened as it seemed, by some unexplained regrets, "This life is all a dream! all a dream!" And so indeed it doubtless seemed to him, as he stood within the shadow of the descending curtain, and recalled from the mists of by-gone years, in dreamy retrospection, the boyhood aims and after trials, struggles and disappointments which, in natural course, make up the sum of our earthly experiences.

Nor, after all, was he so very wide of the mark; for when we are called upon to put off the material and take on the spiritual, what is there left to us of the gleanings of a lifetime, but memory—memory which lives forever in the past, and whose best and brightest accumulations are the unspeakable treasures of the soul.

But it is of dreams, those ever varying pictures flashed upon the brain when the ordinary senses resign their mastery to the more delicate influences of the spirit, that we would particularly direct the attention of our readers. What is that which, under such conditions, makes use of the mental faculties, causing the brain to continue its activity without fatigue, and oftentimes, without consciousness? What is it that sees, hears, takes note, and calculates, or what is equally to the point, invents, imagines and creates, when the vital forces are suspended in a death-like state of insensibility?

The materialist has never been able to answer this question satisfactorily, even to himself; but to one far in his advance, by having accepted the sublime truth of man's dual existence, the union within himself, of the spiritual and the physical; that man is now and here, a spirit inhabiting a physical organism adapted to this, his initial stage of developement, it becomes comparatively easy of solution, for he is able to perceive in respect to dreams, as in all natural things, the intelligently ordered elements of being, acting in accordance with divine law.

We have said that during the process of dreaming, the superinducing intellligences, make use of the brain of the sleeper, correlatively. We are aware that this has been a disputed point with those who have given the subject unusual attention, among the more prominent of whom, may be mentioned the well known philosopher Kant, but later inquirers, whose facilities of investigation were quite extraordinary, have been able to show quite conclusively that even in dreams the mind and brain act in correspondence.

Sir Astly Cooper had a patient whose skull being imperfect, admitted of his examining the movements of the brain, concerning which he says, " I distinctly saw that the pulsation of the brain was regular and slow, except when he was agitated by some opposition to his wishes, and directly the blood was sent with increased force to the brain, the pulsation became frequent and violent."

A parallel case of a sleeping patient is mentioned by several authors. The subject was a female, who had lost a large portion of the skull and *dura mater*, in a neglected attack of *lues venerea.* " When she was in a dreamless sleep her brain was motionless ; when her sleep was imperfect and she was agitated by dreams, her brain protruded from the cranium ; in vivid dreams, *repeated as such by herself*, the protrusion was considerable ; and when perfectly awake, especially if engaged in active thought or sprightly conversation, it was greater still."

These parallel examples are sufficient to show that the mind acts upon the brain in dreams, during the period of sleep, precisely as it does in a state of wakefulness and activity, and in a corresponding degree, depends upon it as a means of either direct or symbolic expression, and what is still more remarkable, the uncompleted efforts of the intellect, are not unfrequently taken up and carried forward in dreams with an energy and skill surpassing its normal achievements.

Tartini's " Devil's Sonata," is a famous example of the exercise of this power. The great composer had endeavored in vain to satisfactorily finish his work, but the inspirarion upon which he depended appeared to have died out, and after repeated failures, he abandoned the task in despair. During the night, he heard in a dream, his magnificent work executed to completion on the violin, and upon waking, he at once wrote it down from memory, and christened it as the incidents of his dream suggested, " Devil's Sonata."

Similar to this was the remarkable dream of Coleridge, told in his " Kubla Khan." Being indisposed, an anodyne had been prescribed, from the effects of which he fell asleep in his chair, at the moment he was reading the following words in " Purchas's Pilgrimage " : " Here Kahn Kubla commanded a palace to be built, and a stately garden," etc. The Poet relates that during his sleep he could not have composed less than three hundred lines, the images rising up before him as things, without sensation or consciousness of effort on his part. On awaking he at first rememmbered the whole of his poetic fancy, and instantly began to write down the lines,

" In Xanadu did Kubla Kahn
A stately pleasure dome decree " etc., but being interrupted in this exercise and detained for a considerable time, he lost the thread of his dream and could not afterwards regain it, an experience familiar doubtless, to many of our readers, for the memory of dreams, for the most part, is almost as transitory as the dream itself, and is likely to elude our mental grasp unless seized upon and fixed in the mind, at the moment of waking.

The " Sheperd " of St. Hermas, the " Divine Comedy " of Dante, and " L'Henriade " of Voltaire, are also notable instances of like intellectual achievement in dreams.

" I said in a dream," writes Voltaire, " things which I could scarcely have said when awake. I must therefore have had thoughts and reflections in spite of myself, and without having taken the least part in them. I had neither will nor liberty, and yet I associated my ideas with propriety and sometimes with genius."

The late Doctor Samuel B. Brittan, so justly distinguished for his learning and eloquence, was at an early period of his life, mainly devoted to lecturing in public. At this time it was no uncommon thing for him to hold forth in his sleep, upon some engrossing theme, with great fervor and clearness. These nocturnal discourses, if uninterrupted, covered the field of argument, from premises to conclusion, with a method and arrangement of parts which, only reason and contemplation are able to achieve ; yet the Doctor remembered nothing of it himself, and had it not been for the affirmance of certain privileged members of his family, he might and probably would have ignored his participation in any such exercise.

A further evidence of this co-operation of mind and brain in sleep, is found in the ability which some persons have to awake at any predetermined hour of the night, thus showing that the will maintains its hold, and gives direction to the human battery, after all its forces appear to have been temporarily suspended.

It is contended by many that it is only possible to dream when in a restless or disturbed state of mind; that when in sound sleep the mind is dormant, and wholly inactive; but whilst no direct proof can be brought to bear upon this point there is an accumulation of circumstantial evidence which would lead to a different conclusion. Though it be true that in a restless, half asleep and half awake state, we are enabled to recall our dreams with more or less clearness, it only goes to show that we are then enabled to make use of faculties sufficiently alert to fix in memory the imaginative adventures of the night, but none even the wisest, are able to demonstrate that the *mind* ever *sleeps*, although it may temporarily relinguish its hold upon the human mechanism by means whereof it is wont sometimes to express itself in a manner independently of it. Such would seem to be the case in the very many well attested instances of "doubles" or what the Germans call "doppelgangers," wherein the intangible resemblance of one still on this side of life, has been known to present itself in a most inexplicable manner, to some near and dear relative or friend, and been seen and recognized and conversed with, when in fact, the individual thus personified, was known to be not only fast asleep at the time, but also many miles distant.

"When the body sleeps," says Tertullian in his "De Anima," "it takes its own peculiar refreshment, but that refreshment, not beiug adapted to to the soul, which does not rest, she during the inactivity of the bodily members, employs her own." The same writer expreses his conviction that future honors, dignities, medical remedies, thefts and treasure, have been revealed by dreams, and certainly, the proof is not wanting to amply sustain the views of this emnient author. It is quite impossible to reconcile these mysterious phenomena, with any mere materialistic theory, or to explain them upon any other hypothesis than the one which concedes the union of the two elements, spirit and matter, in the human organism, and the ability of the former to control the action of the brain in its periods of rest or sleep.

We extract the following from an article by a correspondent of the *Spectator* (Vol. 12, No. 593, A. D. 1714) which appears to us to present a proper view of the subject.

"I see no reason why we should neglect to examine those imaginary scenes we are presented with in sleep, only because they have less reality in them than our waking meditations. A traveler would bring his judgment in question, who should despise the directions of his map for want of real roads in it, because here stands a dot instead of a town, or a cipher instead of a city ; and it must be a long day's journey to travel through two or three inches. Fancy in dreams gives us much such another landscape of life as that does of countries ; and, though its appearances may seem strangely jumbled together, we may often observe such traces and footsteps of noble thoughts, as, if carefully pursued, might lead us nto a proper path of action. There is so much rapture and ectasy in our fancied bliss, and something so dismal and shocking in our fancied misery, that, though the inactivity of the body has given occasion for calling sleep the image of death, the briskness of the fancy affords us a strong intimation of something within us that can never die."

The last quoted sentence of this old-time writer, seems to us to express a conviction that arises naturally, and almost inevitably from an enlightened view of the subject. (*To be continued.*)

